# Southern Medical College, Atlanta

**Published:** 1884-02-20

**Authors:** 


### EDITORIALS AND MISCELLANEOUS.

### *SOUTHERN MEDICAL COLLEGE, ATLANTA.*

The course of lectures in the above Institution during the present season has been thorough and satisfactory, and delivered to a large and intelligent class. The prospect that here, in the centre of the Southern States, a great and efficient school of medicine is being built up is most promising, and is, doubtless, very satisfactory to the profession throughout the entire South. There can no longer be any excuse for our students of medicine to go north to procure a thorough medical education, as they can find here every facility, and at less cost than can be procured abroad.

The commencement exercises of the above school will be published in our next, or March number.

